# Oral post-treatment supplementation with a combination of glutamine, citrulline, and antioxidant vitamins additively mitigates jejunal damage, oxidative stress, and inflammation in rats with intestinal ischemia and reperfusion

**DOI:** 10.1371/journal.pone.0298334

**Published:** 2024-02-02

**Authors:** Yu-Wen Chiu, Chien-Hsing Lee, Hui-Chen Lo

**Affiliations:** 1 Department of Nutritional Science, Fu Jen Catholic University, New Taipei City, Taiwan; 2 Lee’s Endocrinology Clinic, Pingtung City, Pingtung County, Taiwan; 3 School of Chinese Medicine, College of Chinese Medicine, China Medical University, Taichung, Taiwan; 4 Department of Surgery, Division of Pediatric Surgery, China Medical University Children’s Hospital, Taichung, Taiwan; University of Illinois at Chicago, UNITED STATES

## Abstract

**Introduction:**

Intestinal ischemia and reperfusion (IIR) injury is closely associated with oxidative stress. Evidence shows that oral supplementation with glutamine and citrulline alleviates IIR-induced jejunal damage. We investigated the effects of a combination of glutamine, citrulline, and antioxidant vitamins on IIR-induced jejunal damage, oxidative stress, and inflammation.

**Method:**

Male Wistar rats that underwent 60 min of superior mesenteric artery occlusion were orally administered glutamine plus citrulline (GC), vitamin C plus E (CE), or a combination of GC and CE 15 min before and 3, 9, and 21 h after reperfusion. Healthy rats without IIR were used as controls.

**Results:**

After reperfusion for 24 h, rats with IIR showed lower levels of red blood cells, hemoglobin, serum glucose, and jejunal DNA and increased white blood cell counts compared to controls (1-way ANOVA with the least significant difference, *P* < 0.05). The IIR-induced decrease in serum albumin and increase in plasma interleukin-6 and jejunal thiobarbituric acid-reactive substances (TBARS) were significantly reversed by GC and/or CE. The results of the 2-way ANOVA indicated that GC was the main factor that increased jejunal villus height and muscularis DNA, and CE was the main factor that increased jejunal muscularis protein and decreased jejunal proinflammatory cytokine levels and myeloperoxidase activity. In addition, GC and CE are the main factors that decrease plasma proinflammatory cytokine levels and the jejunal apoptotic index.

**Conclusion:**

Oral post-treatment supplementation with glutamine and citrulline, combined with vitamins C and E, may alleviate IIR-induced oxidative stress, inflammation, and jejunal damage.

## Introduction

Intestinal ischemia and reperfusion (IIR) injury is a life-threatening abdominal emergency with a mortality rate > 60% [1]. The causes of IIR include numerous acute pathological conditions such as severe trauma, burns, shock, intestinal torsion, mesenteric thromboembolism, cardiopulmonary resuscitation, and small bowel transplantation [2]. IIR injury involves two stages of insults: blood flow obstruction-induced intestinal disruption due to a lack of oxygen and nutrients, and blood restoration-caused tissue damage due to the release of reactive oxygen species (ROS), inflammatory mediators, and harmful substances [3]. This 2-hit injury eventually leads to a systemic inflammatory response syndrome (SIRS), multiple organ failure (MOF), and even death [1–3]. So far, there are no clinical practice guidelines for the treatment of IIR. Clinical treatment strategies commonly involve a combination of surgery to restore blood flow and medical interventions such as fluid resuscitation, vasodilators, thrombolytics, anticoagulants, antibiotics, anti-inflammatory agents, antioxidants, and nutritional support, to prevent blood clotting and deleterious complications [4].

Disruption of the mucosal barrier is a characteristic feature of IIR injury that contributes to increased intestinal permeability, inflammation, tissue damage, and the potential for systemic complications [5]. Preserving and restoring the integrity of the mucosal barrier is crucial to minimize the harmful effects of IIR injury. Various amino acids, including arginine, glutamine, and citrulline, have been known for their trophic and cytoprotective effects on gut mucosal mass and integrity in IIR animals [6–8]. Evidence indicates that oxidative stress plays a key role in the pathogenesis of IIR injury [3]. For example, pretreatment with antioxidant vitamins, such as vitamins C and E, showed protective effects against IIR-induced intestinal morphological lesions and neutrophil infiltration, increased the end products of lipid peroxidation, and decreased glutathione and antioxidant enzyme activity, including superoxide dismutase, glutathione peroxidase, and catalase [9–12].

Various therapeutic strategies have been proposed to prevent IIR injury progression and manage its complications [4]. In this study, we focused on providing glutamine, a major fuel source for enterocytes and immunocytes [7]. and citrulline, an endogenous amino acid produced from glutamine in enterocytes. Citrulline serves as a source of *de novo* arginine synthesis, aiming to preserve the jejunal barrier [8, 13]. Nitric oxide (NO), derived from arginine, is a major mediator of mucosal cell injury during reperfusion [14]. In a double-blind, randomized, placebo-controlled crossover study, healthy volunteers who received oral citrulline supplementation showed increased plasma arginine and nitric oxide (NO) levels and augmented NO-dependent signaling without causing excess NO-associated adverse effects [15]. In this study, we also focused on alleviating IIR-induced oxidative stress and inflammation by providing antioxidant vitamins, such as vitamin C and E after the ischemia and before the reperfusion.

Our rationale for combining amino acids and antioxidant vitamins is twofold: to provide essential nutrients/fuel to enterocytes and to attenuate IIR-induced oxidative stress. Additionally, our study diverged by employing post-treatment supplementation, a more clinically relevant approach for IIR. Our hypothesis is the first to suggest that, in IIR, post-treatment with a combination of amino acids and antioxidant vitamins may have a complementary effect in enhancing jejunal mucosal integrity, reducing oxidative stress, and alleviating inflammation compared to individual supplementation. Therefore, using a rat model with IIR injury, we investigated the effects of a combination of glutamine, citrulline, and vitamins C and E on jejunal morphology, inflammatory responses, and redox status.

## Materials and methods

### Animals, experimental design, and surgical procedure

Male Wistar rats (BioLASCO Taiwan Co., Ltd., Taipei City, Taiwan), initially weighing 250 grams (7 weeks old), were acclimated to the animal facility with free access to water and a chow diet (5001 Laboratory Rodent diet, Labdiet®, Richmond, IN) in a room maintained at 21 ± 2°C and humidity 55% ± 10% with a 12:12-h light-dark cycle for 1 week.

After overnight fasting, the rats were divided into five groups: a normal control (NC) group (n = 13) and four groups with IIR injury. Rats in the four IIR groups (n = 17 per group) were anesthetized by intramuscular injection with ketamine (80 mg/kg) and xylazine (8 mg/kg), underwent a 60-minute occlusion of the superior mesenteric artery (SMA) using microvascular clamps to induce the intestinal ischemia, and then experienced 24 h of reperfusion before being euthanized, following the procedure outlined in the study of Lai et al. [8]. Forty-five minutes after the ischemia, i.e., 15 minutes before the reperfusion, and 3, 9, and 21 h after the reperfusion, rats were orogastrically administered 200 μl of starch pastes (5% w/v), supplemented with one-, one-, six-, and one-eighth of the daily dose of supplements, respectively [8].

The animal use protocol was reviewed and approved by the Institutional Animal Care and Use Committee (IACUC) of Fu Jen Catholic University, New Taipei City, Taiwan (# FJU-A9727).

**Fig 1:**
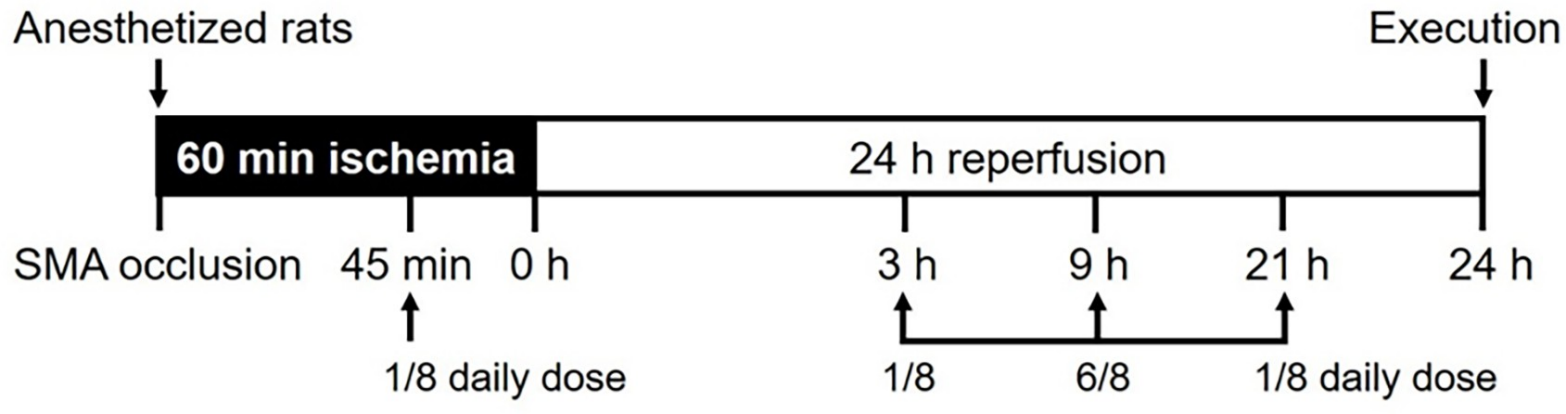
Experimental scheme. After being anesthetized, rats were suffered with the occlusion of the superior mesenteric artery (SMA) for 60 min and with 24 h of reperfusion before the execution. Supplementations of vehicle (casein 2 g/kg/day), glutamine (0.834 g/kg/day) plus citrulline (1 g/kg/day), vitamin C (200 mg/kg/day) plus vitamin E (100 mg/kg/day), or combination of glutamine, citrulline, and vitamins were orogastrically administered at 15 min before, i.e., 45 min after the ischemia, and 3, 9, and, 21 h after the reperfusion, as shown in arrows. Supplementations were mixed in 200 μl of starch pastes (5% w/v) with indicated daily doses.

The supplements administered included vehicle (IIR group), glutamine plus citrulline (GC group), vitamin C plus E (CE group), and a combination of glutamine, citrulline, and vitamins C and E (GC+CE group). The doses of glutamine (0.834 g/kg/day) and citrulline (1 g/kg/day) were determined based on the studies by Osowska et al. [13] and Lai et al. [8] The doses of antioxidant vitamins, namely vitamin C (200 mg/kg/day) and E (α-tocopherol, 100 mg/kg/day), were in accordance with the study by Kacmaz et al. [16]. Each of the four starch pastes, corresponding to the different supplement groups, contained an equal amount of nitrogen (i.e., 5.708 mmol of N/kg/day), as specified in the study by Osowska et al. [13] and an equivalent protein content achieved by adding casein (2 g/kg/day) as a vehicle to control for the protein content. The time intervals and doses of amino acid supplementation were selected to align with a three-meal-a-day food intake pattern after adjustment for the time intervals [8]. The experimental design is illustrated in Fig 1.

Twenty-four hours after the reperfusion, rats were anesthetized with an overdose of pentobarbital (75 mg/kg, i.p.) and subsequently euthanized via cardiac puncture under unconscious conditions. Body weight was recorded and blood samples were obtained. Subsequently, serum, plasma, and whole blood were isolated for further assays. Organs including the lungs, heart, liver, kidneys, spleen, thymus, and gastrocnemius muscles were dissected and weighed, with the results documented. The entire small intestine was carefully removed and placed on an ice-cold plate. The segment extending from the oral 10 cm from the stomach to the distal 15 cm from the cecum end was considered as the jejunum.

The final sample size in the NC, IIR, GC, CE, and GC+CE groups was 13, 17, 16, 15, and 15, respectively, with the loss of 5 rats for the intragastric administration.

### Complete blood counts (CBC) and serum biochemistry parameters

A complete blood count (CBC), including the numbers of red blood cells (RBC), white blood cells (WBC), platelets, hematocrit percentages, and hemoglobin levels in the whole blood, were determined using a hematology analyzer (GEN-S System 2, Beckman Coulter, Inc., CA). The concentrations of serum biochemical parameters, including glucose, albumin, triglycerides, cholesterol, blood urea nitrogen (BUN), glutamic pyruvic transaminase (GPT), and glutamic oxaloacetic transaminase (GOT), were measured using an automatic analyzer (Hitachi 7150, Tokyo, Japan).

### Plasma and jejunal levels of amino acids, vitamin C, and vitamin E

The levels of amino acids, including glutamine, ornithine, citrulline, and arginine, in both plasma and jejunal samples were determined using high-performance liquid chromatography (HPLC) with the PICO-TAG method (Millipore, Merck KGaA, Darmstadt, Germany). Alpha-aminobutyric acid served as the sample preparation internal standard, following the methodology described by Bidlingmeyer et al. [17].

Additionally, the levels of vitamins C and E in both plasma and jejunal samples were determined using the methods outlined in the studies by Rumelin et al. [18] and Barbas et al. [19], respectively. For vitamin C measurements, plasma and jejunal samples underwent deproteinization using perchloric acid. After centrifugation, the supernatant was subjected to HPLC with UV absorbance at 245 nm. Meanwhile, for vitamin E measurements, plasma and jejunal samples were extracted with n-hexane, dried using nitrogen evaporation, re-dissolved in a chloroform-methanol mixture (1:3, v/v), and introduced into the HPLC system with UV absorbance at 295 nm.

### Plasma and jejunal levels of inflammatory mediators and lipid peroxidation

The plasma concentrations and jejunal contents of tumor necrosis factor (TNF)-α, interferon (IFN)-γ, and interleukin (IL)-6 were measured using commercially available enzyme-linked immunosorbent assays (ELISA; R&D Systems, Inc., MN, USA). Additionally, the levels of nitrate/nitrite (NOx), serving as an indirect indicator of NO, were determined using a commercial colorimetric kit (Cayman Chemical, Ann Arbor, MI, USA). All analytical procedures were conducted in accordance with the manufacturer’s instructions.

The end products of lipid peroxidation, thiobarbituric acid reactive substances (TBARS), in both plasma and jejunum were determined using the method outlined by Ohkawa et al. [20]. The fluorescence intensity of TBARS was determined at excitation and emission wavelengths of 515 and 550 nm, respectively. TBARS levels were quantified using a 1,1,3,3-tetramethoxypropane (TMP) standard curve. Jejunal levels of TNF-α, IFN-γ, IL-6, NOx, and TBARS were adjusted for jejunal weight.

### Jejunal morphological and mass changes

To observe morphological changes in the jejunum, a 1 cm segment from the central portion was fixed in 10% buffered formalin for routine paraffin embedding. The resulting 4 μm paraffin embedded sections were deparaffinized, stained with hematoxylin and eosin (H&E), and morphometrically measured under a light microscope (Nikon Eclipse TE2000-U, Nikon Inc., Melville, NY). For each rat, at least five villus-crypt axes were measured to determine jejunal villus height, crypt depth, and muscularis thickness.

To assess wet and dry mucosal and muscularis weights, a 3 cm segment of the jejunum near the center was collected. The mucosa was scraped using a glass slide, and both mucosa and muscularis were weighed to obtain the wet weight. Subsequently, they were oven-dried at 80°C for 48 h to obtain the dry weight. Another 3 cm segment near the center was scraped for protein measurement (Bicinchoninic acid protein assay; Pierce Chemical Co. Rockford, IL, USA) and DNA content [21].

### Jejunal apoptotic status and myeloperoxidase (MPO) activity

To identify the apoptotic cells in the jejunum, 4 μm paraffin-embedded sections were stained using the terminal deoxynucleotidyl transferase-mediated dUTP-biotin nick end labeling (TUNEL) technique. A commercially available kit (Hoffmann-La Roche Inc., Branford, CT) was employed following the manufacturer’s instructions. The jejunal sections were counterstained with 4’-6-Diamidino-2-phenylindole (DAPI) for nuclei visualization. The total number of cells exhibiting a green fluorescent signal on TUNEL and a blue fluorescent signal on DAPI (excited by UV light) was quantified using the Image-Pro Plus software (version 6.0; Media Cybernetics, Inc., Silver Spring, MD, USA). The apoptotic index (AI) of the jejunum was calculated as the number of TUNEL-positive cells divided by the total number of DAPI-positive cells in the villi [22].

To assess MPO activity, jejunal samples were pre-treated with hexadecyltrimethylammonium bromide (Sigma-Aldrich Inc., St. Louis, MO) and then reacted with o-dianisidine and hydrogen peroxide (H_2_O_2_). The absorbance of the resulting mixture was measured at 450 nm immediately and after 5 min. MPO activity was calculated using the method outlined by Krawisz et al. [23].

### Statistical analysis

Values are reported as mean ± SEM. All treatment groups were compared using a one-way analysis of variance (ANOVA). Fisher’s least significant difference (LSD) test was served as a *post hoc* analysis to compare differences between groups when one-way ANOVA indicated an overall significant group effect at *P* < .05. Additionally, a 2-way ANOVA was employed to determine the main effects of GC and CE, as well as the interaction between GC and CE on each parameter among the IIR, GC, CE, and GC+CE groups. Data were analyzed using SAS Version 9.4 (SAS Institute, Inc., Cary, NC, USA). *P* value < .05 was considered statistically significant. A *post hoc* power calculation was performed using the statistical power analysis G*Power 3.1 [24]. For the four IIR groups, ten rats were necessary for ≥ 80% power at a 5% significance level.

## Results

### Body weight and organs and tissues weights

There were no significant differences in body weight and the weights of the heart, lungs, kidneys, and gastrocnemius muscle among the groups. However, the liver weights were significantly greater in the IIR group (10.42 ± 0.24 g) than in the NC group (9.26 ± 0.58 g). Additionally, the weights of the thymus and spleen were significantly lower in the IIR group (0.47 ± 0.02 and 0.66 ± 0.04 g, respectively) than in the NC group (0.61 ± 0.04 and 0.81 ± 0.04 g, respectively). The spleen weights were significantly greater in the GC and CE groups (0.79 ± 0.03 and 0.78 ± 0.04 g, respectively) than in the IIR group.

The results of the two-way ANOVA showed that neither supplementation of glutamine plus citrulline nor vitamin C plus E was the main factor altering the body weights or the weights of the heart, lung, liver, gastrocnemius muscle, and thymus in IIR rats. However, supplementation with glutamine and citrulline was the main factor that increased kidney weight. In addition, there was an interaction between supplementation with glutamine plus citrulline and vitamin C plus E on spleen weights, as evidenced by the significantly increased spleen weights in rats treated with either glutamine plus citrulline or vitamin C plus E, but not with their combination.

### Hematology and serum biochemistry parameters

The results of CBC and serum concentrations of the biochemical parameters are shown in Table 1. The IIR group exhibited significantly decreased circulating numbers of RBC and platelets (791 ± 20 ×10^3^/μl), hematocrit percentages (39.6 ± 0.79 ml/%), and hemoglobin levels, along with increased circulating numbers of WBC, compared to the NC group (platelet, 967 ± 34 ×10^3^/μl; hematocrit, 43.7 ± 0.5 ml/%). Neither glutamine plus citrulline nor vitamin C plus E supplementation was the main factor altering CBC among the four IIR groups (two-way ANOVA, *P* < .05).

**Table 1:** Complete blood cell counts and serum biochemical parameters.

	RBC	WBC	Hemoglobin	Glucose	Albumin	BUN	GOT	GPT
Group	10^6^/μL	10^3^/μL	g/100 mL	mg/100 mL	g/100 mL	mg/100 mL	U/L	U/L
NC	6.87 ± 0.07	4.55 ± 0.19	14.3 ± 0.2	168.6 ± 13.3	3.09 ± 0.03	16.1 ± 1.5	121 ± 12	33.5 ± 2.1
IIR	6.47 ± 0.14*	5.46 ± 0.31*	13.3 ± 0.3*	141.4 ± 4.4*	2.51 ± 0.06*	27.5 ± 1.1*	564 ± 52*	129.0 ± 14.9*
GC	6.27 ± 0.10*	5.69 ± 0.18*	13.1 ± 0.2*	139.3 ± 3.5*	2.69 ± 0.04*†	32.8 ± 3.0*	485 ± 26*	102.5 ± 7.0*
CE	6.43 ± 0.15*	5.64 ± 0.31*	13.3 ± 0.2*	145.4 ± 4.5*	2.77 ± 0.05*†	25.1 ± 1.6*	476 ± 37*	90.2 ± 7.3*†
GC+CE	6.61 ± 0.13	5.49 ± 0.26*	13.7 ± 0.2	145.1 ± 5.1*	2.77 ± 0.06*†	30.2 ± 1.7*	464 ± 25*†	94.8 ± 6.4*†
GC	NS	NS	NS	NS	NS	0.011	NS	NS
Main effects and interactions in the IIR, GC, CE, and GC+CE groups (2-way ANOVA)								
CE	NS	NS	NS	NS	0.003	NS	NS	0.027
Interaction	NS	NS	NS	NS	0.014	NS	NS	NS

Values are mean ± SEM, n = 13, 17, 16, 15, and 15 in the NC, IIR, GC, CE, and GC+CE groups, respectively. Values with the symbols * and † significantly differ from the NC and IIR groups, respectively (P < .05, 1-way ANOVA with the least significant difference). Values of 2-way ANOVA are P-values for the main effects of GC and CE and the interaction between GC and CE in the IIR, GC, CE, and GC+CE groups. BUN, blood urea nitrogen; GOT, glutamic oxaloacetic transaminase; GPT, glutamic pyruvic transaminase; NS, not significant; RBC, red blood cells; WBC, white blood cells.

There were no significant differences in the serum concentrations of triglycerides, cholesterol, or creatinine between the groups. However, the serum concentrations of albumin and glucose were significantly decreased, and those of BUN, GOT, and GPT were significantly higher in the IIR group than in the NC group. The IIR-induced decrease in serum albumin concentration was significantly increased by supplementation with glutamine plus citrulline and/or vitamin C plus E, as evidenced by higher concentrations in the GC, CE, and GC+CE groups than in the IIR group. In addition, the IIR-induced increase in serum concentrations of GOT partially but significantly decreased in animals supplemented with a combination of glutamine, citrulline, and vitamin C plus E. Meanwhile, the serum concentration of GPT decreased approximately 25% in those supplemented with vitamin C plus E, with or without glutamine plus citrulline. Supplementation with glutamine plus citrulline was the main factor that increased serum BUN concentration, while supplementation with vitamins C and E was the main factor that increased serum albumin and decreased serum GPT in IIR rats.

### Amino acid levels in the plasma and jejunum

The results for arginine metabolism-associated amino acids in plasma and jejunum are detailed in Table 2. In plasma, concentrations of arginine, but not glutamine, ornithine, or citrulline, were significantly lower in the IIR group than in the NC group. Citrulline and arginine concentrations in plasma were significantly higher in the GC and GC+CE groups than in the IIR group. Supplementation with glutamine and citrulline was the main factor increasing plasma ornithine, citrulline, and arginine concentrations, while supplementation with vitamins C and E was the main factor decreasing plasma citrulline and arginine concentrations in IIR rats. The results of 2-way ANOVA indicated an interaction between supplementation with glutamine plus citrulline and vitamin C plus E on plasma citrulline and arginine concentrations.

**Table 2:** Glutamine, ornithine, citrulline and arginine levels in the plasma and jejunum.

	Plasma					Jejunum			
	Glutamine	Ornithine	Citrulline	Arginine		Glutamine	Ornithine	Citrulline	Arginine
Group	μmol/L	μmol/L	μmol/L	μmol/L		nmol/g	nmol/g	nmol/g	nmol/g
NC	493 ± 23	392 ± 30	122 ± 4	312 ± 14		3.24 ± 0.25	1.88 ± 0.14	0.82 ± 0.03	11.1± 0.74
IIR	513 ± 7	429 ± 21	106 ± 4	252 ± 12*		2.66 ± 0.12*	1.65 ± 0.12*	0.80 ± 0.02	9.1 ± 0.4
GC	462 ± 21	481 ± 21	350 ± 46*†	444 ± 22*†		2.76 ± 0.10*	1.66 ± 0.12	1.15 ± 0.06*†	9.8 ± 0.4
CE	480 ± 19	405 ± 20	107 ± 3	285 ± 13		2.87 ± 0.14	1.70 ± 0.10	0.90 ± 0.04	10.1 ± 0.5
GC+CE	452 ± 21	443 ± 16	220 ± 17*†	334 ± 9†		2.91 ± 0.16	1.96 ± 0.09†	0.98 ± 0.03*†	10.5 ± 0.7
Main effects and interactions in the IIR, GC, CE, and GC+CE groups (2-way ANOVA)									
GC	NS	0.028	< 0.001	< 0.001		NS	NS	< 0.001	NS
CE	NS	NS	0.011	0.128		NS	NS	NS	NS
Interaction	NS	NS	0.010	< 0.001		NS	NS	0.001	NS

Values are mean ± SEM, n = 13, 17, 16, 15, and 15 in the NC, IIR, GC, CE, and GC+CE groups, respectively. Values with the symbols * and † significantly differ from the NC and IIR groups, respectively (P < .05, 1-way ANOVA with the least significant difference). Values of 2-way ANOVA are P-values for the main effects of GC and CE and the interaction between GC and CE in the IIR, GC, CE, and GC+CE groups. NS, not significant.

In the jejunum, the amounts of glutamine and ornithine per gram of tissue was significantly lower in the IIR group than in the NC group, and the amount of ornithine was significantly higher in the GC+CE group than in the IIR group. The amount of jejunal citrulline was significantly greater in the GC and GC+CE groups than in the IIR and NC groups. There was no significant difference in the amount of jejunal arginine between the groups. In addition, supplementation with glutamine and citrulline was the main factor increasing the amount of jejunal citrulline, interacting with vitamins C and E supplementation, as indicated by the interfered increase in the combination supplementation.

### Vitamin C, vitamin E, and TBARS levels in the plasma and jejunum

To assess the impact of IIR and IIR supplementation on redox status, the levels of vitamin C, vitamin E, and TBARS in plasma and jejunum were determined, and the results are summarized in Table 3. No significant differences were observed in plasma levels of vitamin C, vitamin E, or TBARS between the IIR and NC groups. However, the CE and GC+CE groups displayed 2.2- and 3.1-fold increases, respectively, in plasma vitamin E concentrations compared with the IIR group and 3.0- and 4.2-fold increases, respectively, when compared with the NC group. The GC+CE group exhibited significantly lower plasma TBARS concentrations than the IIR and NC groups. Results from the 2-way ANOVA indicated that supplementation with glutamine plus citrulline was the main factor increasing plasma vitamin E concentration and decreasing plasma TBARS concentration. Furthermore, vitamin C plus E was the main factor increasing plasma vitamin C and E concentrations.

**Table 3:** Vitamin C, vitamin E, and TBARS levels in the plasma and jejunum.

	Plasma				Jejunum		
	Vitamin C	Vitamin E	TBARS		Vitamin C	Vitamin E	TBARS
Group	μg/mL	μg/mL	nmol/mL		μg/g	μg/g	nmol/g
NC	8.5 ± 1.0	1.92 ± 0.32	3.20 ± 0.12		193 ± 17	10.5 ± 1.2	19.2 ± 1.2
IIR	10.4 ± 0.5	2.64 ± 0.34	2.87 ± 0.16		175 ± 10	6.5 ± 0.5	25.1 ± 2.5*
GC	9.0 ± 0.3	3.23 ± 0.30	2.77 ± 0.13*		215 ± 8	7.8 ± 0.6	16.7 ± 1.2†
CE	12.3 ± 1.0*	5.79 ± 0.65*†	3.02 ± 0.19		348 ± 29*†	15.4 ± 1.5†	16.3 ± 1.8†
GC+CE	12.1 ± 0.6*	8.10 ± 0.68*†	2.34 ± 0.09*†		369 ± 16*†	31.4 ± 5.0*†	17.6 ±1.4†
Main effects and interactions in the IIR, GC, CE, and GC+CE groups (2-way ANOVA)							
GC	NS	0.008	0.018		NS	0.001	NS
CE	< 0.001	< 0.001	NS		< 0.001	< 0.001	0.037
Interaction	NS	NS	NS		NS	0.005	0.012

Values are mean ± SEM, n = 13, 17, 16, 15, and 15 in the NC, IIR, GC, CE, and GC+CE groups, respectively. Values with the symbols * and † significantly differ from the NC and IIR groups, respectively (P < .05, 1-way ANOVA with the least significant difference). Values of 2-way ANOVA are P-values for the main effects of GC and CE and the interaction between GC and CE in the IIR, GC, CE, and GC+CE groups. NS, not significant; TBARS, thiobarbituric acid reactive substances.

In the jejunum, no significant difference was observed in the vitamin C or E content between the IIR and NC groups. However, the jejunal vitamin C content increased by 2- and 2.1-fold, respectively, and the jejunal of vitamin E content increased by 2.4- and 4.8-fold, respectively, in the CE and GC+CE groups when compared with the IIR group. Jejunal TBARS content was significantly higher in the IIR group than in the NC group and was significantly lower in the GC, CE, and GC+CE groups than in the IIR group.

The results of the 2-way ANOVA indicated that supplementation with glutamine plus citrulline was the main factor increasing plasma vitamin E concentration and decreasing plasma TBARS concentration. Vitamin C plus E was the main factor increasing plasma vitamin C and E concentrations. In the jejunum, supplementation with glutamine plus citrulline was the main factor increasing E content, while supplementation with vitamin C plus E was the main factor increasing vitamin C and E content and decreasing TBARS content.

However, interactions were observed between the jejunal contents of vitamin E and TBARS in the context of supplementation with glutamine plus citrulline and vitamin C plus E. For instance, jejunal vitamin E content was synergistically increased in rats with combination supplementation (GC+CE group) compared to rats with amino acid or antioxidant vitamin supplementation (GC or CE group). However, jejunal TBARS content in rats receiving combination supplementation was similar to that in rats receiving amino acid or antioxidant vitamin supplementation.

### Inflammatory mediators in the plasma and jejunum

To assess systemic and local inflammatory responses, TNF-α, IL-6, IFN-γ, and NOx levels in plasma and jejunum were measured, as detailed in Table 4. Plasma concentrations of IL-6 and NOx were significantly increased by approximately 2.8 and 1.9-folds, respectively, while those of TNF-α and IFN-γ were significantly decreased by approximately 30% in the IIR group compared with the NC group. The GC and CE groups exhibited significantly decreased IL-6 and NOx, and the GC+CE group showed significantly decreased IL-6, along with further decreased TNF-α and IFN-γ in plasma compared with the IIR group.

**Table 4:** Proinflammatory cytokine and nitrate/nitrite levels in the plasma and jejunum.

	Plasma					Jejunum			
	TNF-α	IL-6	IFN-γ	NOx		TNF-α	IL-6	IFN-γ	NOx
Group	pg/mL	pg/mL	pg/mL	μmol/L		pg/g	pg/g	pg/g	ng/g
NC	30.7 ± 3.2	27.3 ± 11.3	21.1 ± 3.0	36.8 ± 1.6		504 ± 207	1225 ± 290	501 ± 98	17.3 ± 1.0
IIR	22.1 ± 3.2*	75.5 ± 17.1*	16.2 ± 0.8*	68.0 ± 5.1*		1217 ± 213*	2247 ± 261*	843 ± 110*	17.0 ± 0.5
GC	20.2 ± 1.7*	33.3 ± 8.1†	16.2 ± 1.2*	60.0 ± 3.7*†		1050 ± 148*	2113 ± 204*	991 ± 150*	18.8 ± 0.8
CE	19.2 ± 0.3*	34.5 ± 3.7†	16.1 ± 1.5*	59.8 ± 4.1*†		849 ± 163	1752 ± 180	682 ± 79	17.1 ± 1.1
GC+CE	16.7 ± 0.5*†	30.9 ± 4.9†	9.0 ± 1.3*†	65.5 ± 2.6*		637 ± 115†	1492 ± 153†	533 ± 84	17.7 ± 0.9
Main effects and interactions in the IIR, GC, CE, and GC+CE groups (2-way ANOVA)									
GC	0.001	0.035	0.006	NS		NS	NS	NS	NS
CE	0.004	0.045	0.004	NS		0.028	0.013	0.009	NS
Interaction	NS	NS	0.005	0.035		NS	NS	NS	NS

Values are mean ± SEM, n = 8, 10, 9, 9, and 9 in the NC, IIR, GC, CE, and GC+CE groups, respectively. Values with the symbols * and † significantly differ from the NC and IIR groups, respectively (P < .05, 1-way ANOVA with the least significant difference). Values of 2-way ANOVA are P-values for the main effects of GC and CE and the interaction between GC and CE in the IIR, GC, CE, and GC+CE groups. IFN, interferon; IL, interleukin; NOx, nitrate and nitrite; NS, not significant; TNF, tumor-necrosis factor.

The results of the 2-way ANOVA indicated that supplementation with glutamine plus citrulline and vitamins C and E were the main factors decreasing plasma concentrations of TNF-α, IL-6, and IFN-γ, interacted with plasma IFN-γ and NOx in the IIR rats. For instance, plasma IFN-γ was not significantly altered in the GC and CE groups but was significantly decreased in the GC+CE group compared to the IIR group. Additionally, plasma NOx was significantly decreased in the GC and CE groups but was not significantly altered in the GC+CE group compared to the IIR group.

The levels of TNF-α, IL-6, and IFN-γ per gram of jejunum were significantly increased by approximately 2.4-, 1.8-, and 1.7-fold, respectively, in the IIR group compared to the NC group. However, jejunal levels of TNF-α and IL-6 were significantly lower in the GC+CE group than in the IIR group. There was no significant difference in jejunal NOx content among the groups. Supplementation with vitamins C and E was the main factor decreasing the jejunal content of TNF-α, IL-6, and IFN-γ in IIR rats.

### Morphology and composition of the jejunum

**Fig 2:**
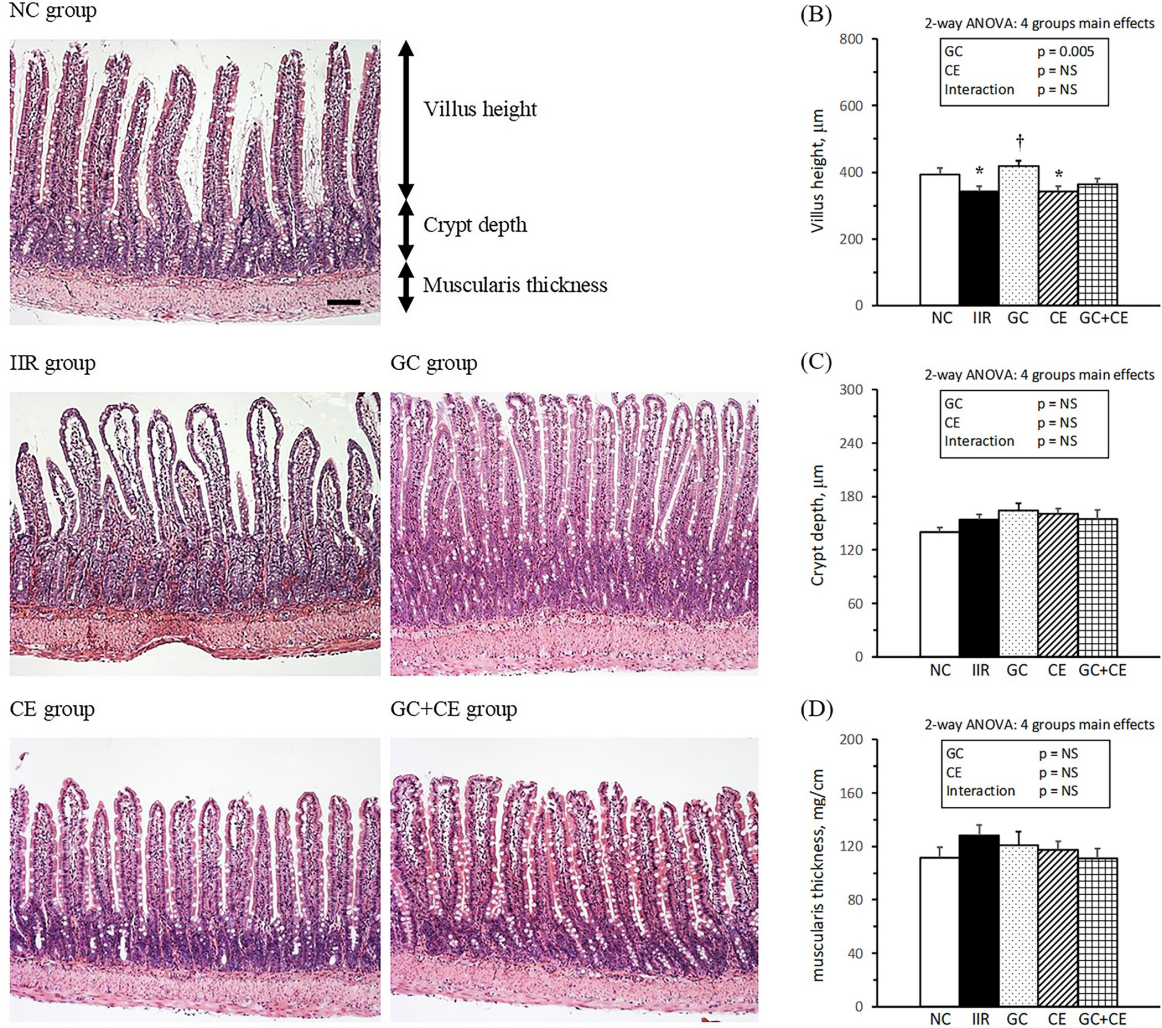
Jejunal histology. Light micrographs of the jejunum stained with hematoxylin and eosin (A) (100×, bars = 100 μm), villus height (B), crypt depth (C), and muscularis thickness (D). Values are mean ± SEM, n = 13, 17, 16, 15, and 15 in the NC, IIR, GC, CE, and GC+CE groups, respectively. Values with the symbols * and † significantly differ from the NC and IIR groups, respectively (*P* < .05, 1-way ANOVA with the least significant difference). Values of 2-way ANOVA are *P*-values for the main effects of GC and CE and the interaction between GC and CE in the IIR, GC, CE, and GC+CE groups. NS, not significant.

Fig 2A illustrates the morphological changes in the jejunum. The IIR group exhibited identifiable mucosal destruction, characterized by a detached epithelial layer from the lamina propria and shortened height in the jejunal villi compared with the NC group. These changes were alleviated in the GC, CE, and GC+CE groups compared with the IIR group. To quantify the morphological changes in the jejunum, villus height, crypt depth, and muscularis thickness were determined, and the results are shown in Fig 2B–2D, respectively.

The IIR group had significantly decreased villus height in the jejunum compared to the NC group. However, the GC group showed a significantly alleviated decrease in villus height compared to the IIR group. Additionally, supplementation with glutamine and citrulline was the main factor increasing jejunal villus height in IIR rats. No significant differences in crypt height or muscularis thickness in the jejunum were found between groups.

**Fig 3:**
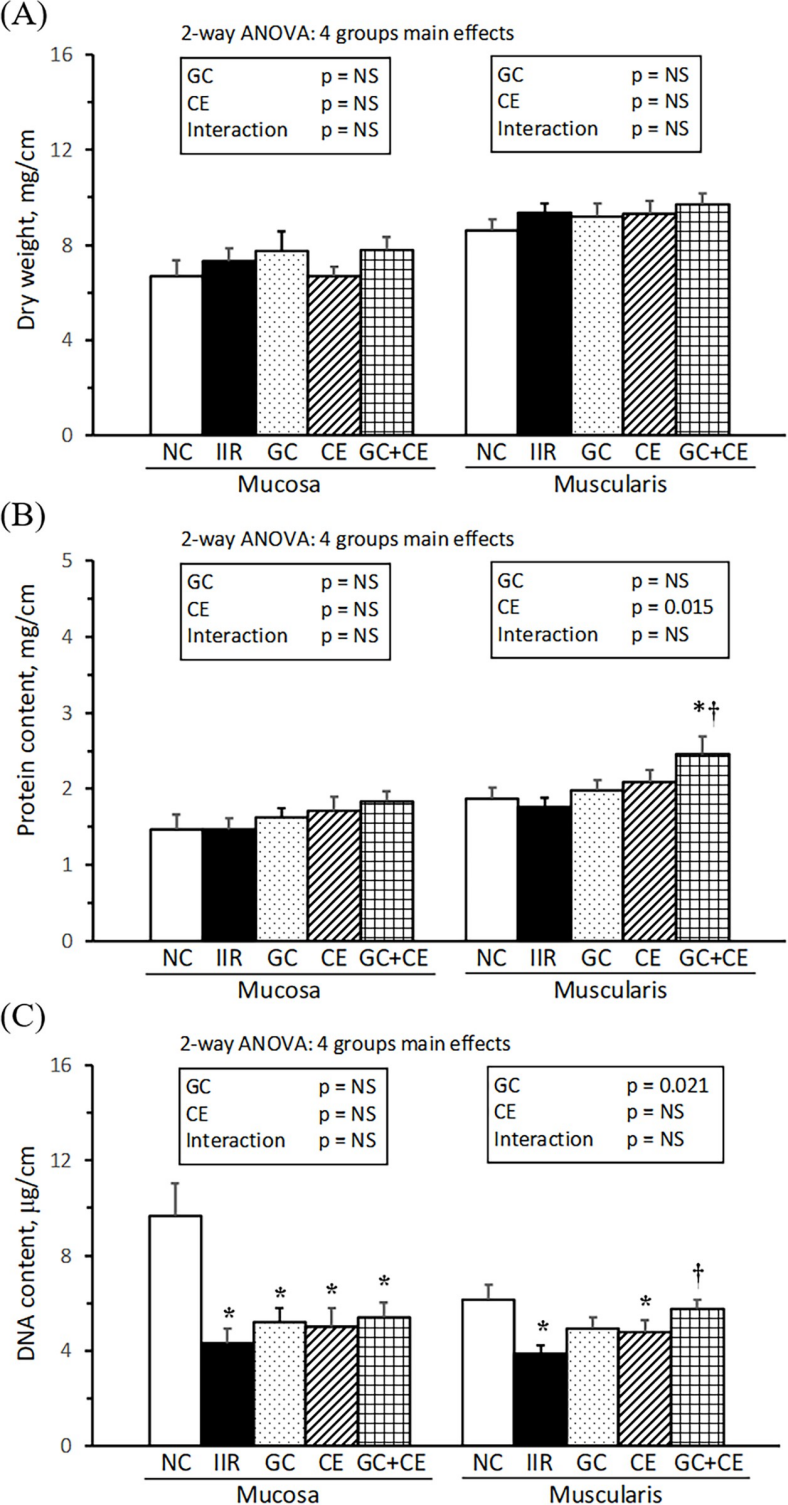
Jejunal mass. Dry weight (A), protein content (B), and DNA content (F) in the jejunal mucosa and muscularis per cm of the jejunum. Values are mean ± SEM, n = 13, 17, 16, 15, and 15 in the NC, IIR, GC, CE, and GC+CE groups, respectively. Values with the symbols * and † significantly differ from the NC and IIR groups, respectively (*P* < .05, 1-way ANOVA with the least significant difference). Values of 2-way ANOVA are *P*-values for the main effects of GC and CE and the interaction between GC and CE in the IIR, GC, CE, and GC+CE groups. NS, not significant.

The wet and dry weights, as well as the protein and DNA contents of the jejunal mucosa and muscularis are presented in Fig 3. Neither the wet weight nor the dry weight of the jejunal mucosa and muscularis differed significantly among the groups. The protein content in the jejunal mucosa and muscularis was not significantly different between the IIR and NC groups; however, the jejunal muscularis protein content was significantly higher in the GC+CE group than in the IIR and NC groups. The DNA content in the jejunal mucosa and muscularis was significantly lower in the IIR group than in the NC group, and that in the jejunal muscularis was significantly higher in the GC+CE group than in the IIR group. Supplementation with vitamin C plus E and glutamine plus citrulline was the main factor increasing the protein and DNA content, respectively, in the jejunal muscularis of IIR rats.

### Jejunal apoptosis

**Fig 4:**
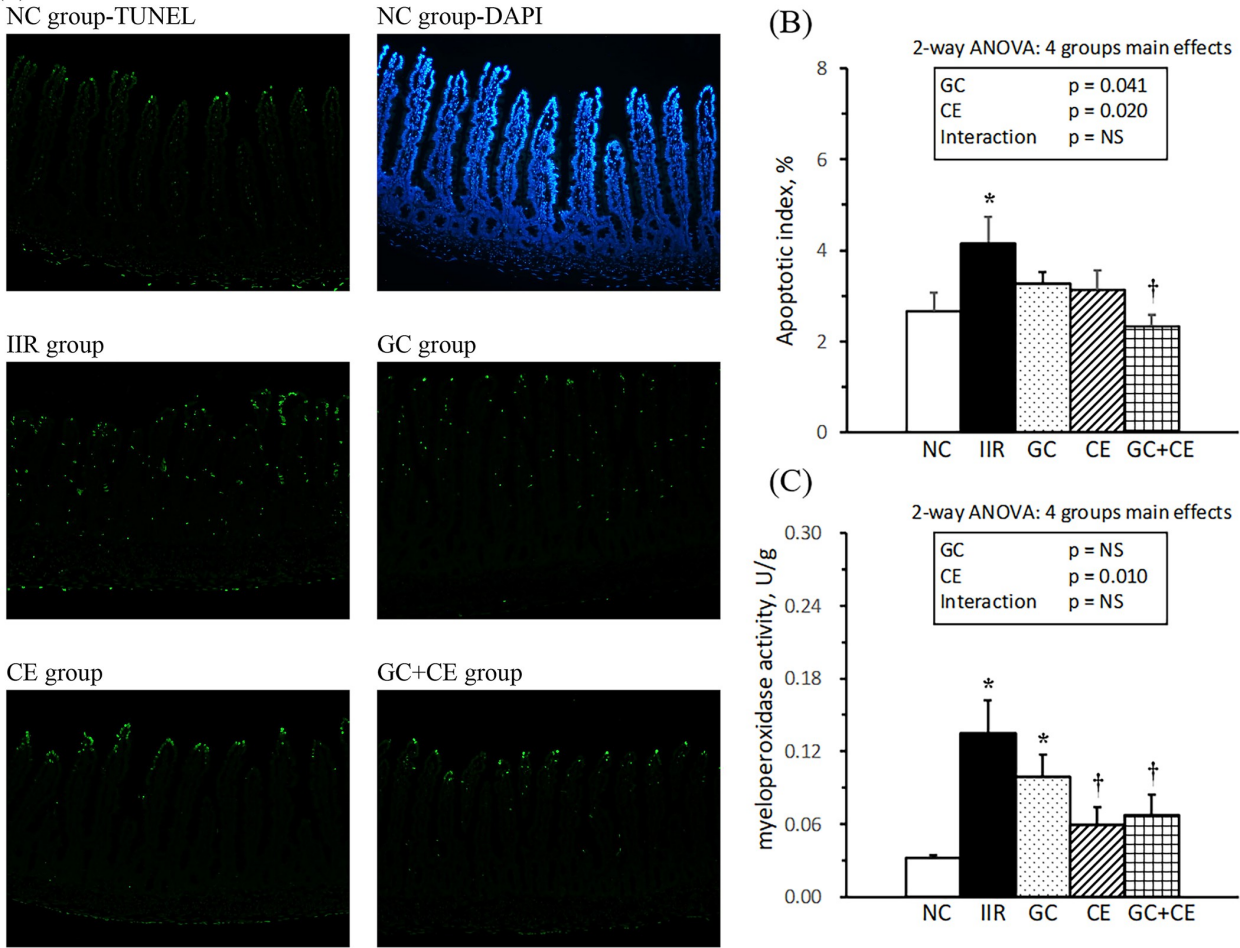
Jejunal apoptosis and myeloperoxidase activity. *In situ* detection of the apoptotic cells using a TUNEL assay with a green fluorescein label in the jejunal villi (A). The total nuclei stained with DAPI are shown in the blue label. The apoptotic index (B) was calculated as the percentage of the TUNEL-positive apoptotic nuclei divided by the DAPI-staining nuclei per 5 villi. The myeloperoxidase activity per gram of the jejunum (C). Values are mean ± SEM, n = 13, 17, 16, 15, and 15 in the NC, IIR, GC, CE, and GC+CE groups, respectively. Values with the symbols * and † significantly differ from the NC and IIR groups, respectively (*P* < .05, 1-way ANOVA with the least significant difference). Values of 2-way ANOVA are *P*-values for the main effects of GC and CE and the interaction between GC and CE in the IIR, GC, CE, and GC+CE groups. NS, not significant.

The results of DNA damage in the jejunum, as determined by *in situ* immunofluorescent TUNEL analysis, are presented in Fig 4A. Green fluorescence (i.e., TUNEL-positive cells) on the villi was more prominent in the IIR group than in the NC group. When the apoptotic index was calculated as the percentage of TUNEL-positive cells per five villi, the IIR group exhibited a significantly higher apoptotic index than the NC group, and the GC+CE group showed a significantly lower apoptotic index than the IIR group (Fig 4B). Supplementation with glutamine plus citrulline and vitamin C plus E was the main factor decreasing the apoptotic index in IIR rats.

MPO activity, an index of activated neutrophils per gram of the jejunum, is shown in Fig 4C. MPO activity was significantly higher in the IIR group than in the NC group and significantly lower in the CE and GC+CE groups than in the IIR group. Supplementation with vitamins C and E was the main factor decreasing MPO activity in the jejunum of IIR rats.

## Discussion

IIR is an emergent condition that involves inadequate blood supply, intestinal damage and dysfunction, and reperfusion-induced injury, including systemic inflammation, multiple organ dysfunction, sepsis, and even death [3]. Evidence shows that antioxidants, anti-inflammatory agents, and substances that maintaining intestinal barrier function may be useful strategies to minimize IIR injury [2, 5]. In this study, we demonstrated that oral post-treatment supplementation with a combination of glutamine, citrulline, vitamin C, and vitamin E may preserve jejunal mass and alleviate oxidative stress and inflammation systemically and locally in an additive and complementary manner.

Using a rat model with 60 min of mesenteric artery occlusion, we mimicked the clinical condition of intestinal ischemia and administered oral supplementation during the perioperative and postoperative periods (15 min before and 3, 9, and 21 h after reperfusion) to provide fuel and modulate the redox status and inflammatory response in enterocytes. After 24 h of reperfusion, the IIR rats without supplementation showed an observably detached epithelial layer from the lamina propria on the jejunal mucosa (Fig 2A), significantly decreased villus height (Fig 2B) and mucosal and muscularis DNA content (Fig 3C). Additionally, there was an increase in the jejunal apoptotic index and neutrophil infiltration, reflected by elevated MPO activity (Fig 4A to 4C). IIR rats also exhibited altered hematology and serum biochemistry parameters, such as decreased RBC, hemoglobin, and albumin levels and increased white blood cell counts and BUN, GOT, and GPT levels (Table 1). Accompanied by increased liver weights and decreased thymus and spleen weights, these results revealed that IIR rats experienced jejunal, liver, and immune dysfunction. Furthermore, the significantly decreased plasma arginine and jejunal glutamine and ornithine (Table 2) imply altered metabolism in arginine-associated amino acids in IIR rats, which aligns with the beneficial effects of arginine on reducing intestinal injury and systemic inflammation in IIR pigs observed in the study of Spanos et al. [25]. Moreover, the increased plasma IL-6 and NOx and jejunal TBARS, TNF-α, and IL-6 (Table 4) indicate that IIR rats were under a condition with systemic and local inflammation. Taken together, these changes confirm that IIR rats suffered from jejunal damage, as well as local and systemic oxidative stress and inflammation, as reported in human clinical and pre-clinical studies [26, 27].

Glutamine, a precursor of nucleotide synthesis and the preferred fuel for enterocytes and immunocytes, has been reported to alleviate IIR-increased endoplasmic reticulum stress and apoptosis in intestinal epithelial cells [7]. Citrulline, a biomarker of intestinal mass and absorptive function, has demonstrated efficacy in ameliorating chronic hypoxia-induced pulmonary hypertension [28] and celiac artery obstruction-induced increases in lipid peroxidation, MPO, and inducible nitric oxide synthase (NOS) activity in the stomach [29]. Our previous study indicated that oral citrulline alleviates IIR-induced jejunal damage and systemic inflammation through the inactivation of NOS and the nuclear factor-κB pathway [8]. In this study, IIR rats supplemented with glutamine plus citrulline exhibited improved jejunal morphological changes and villus height (Fig 2B), along with decreased jejunal TBARS (Table 3) and plasma IL-6 and NOx levels (Table 4), indicating enhanced jejunal integrity, improved redox status, and reduced systemic inflammation. Furthermore, the IIR-induced decrease in plasma arginine levels was reversed by glutamine plus citrulline supplementation (Table 2). These results confirm the beneficial effects of the oral supplemenation of glutamine and citrulline on IIR injury, consistent with our previous study [8].

Antioxidant-based therapies have demonstrated promising results in improving IIR injuries [30]. In IIR rats, vitamin C administration significantly decreased lipid peroxidation, glutathione levels, and mucosal injury in the small intestine [11, 31]. In mice with myocardial ischemia and reperfusion, pretreatment of α-tocopherol, i.e., a fat-soluble vitamin with potent antioxidant activity, significantly decreased cardiac ROS production, neutrophil infiltration, and MPO activity [32]. Pretreatment with vitamins C and E markedly reduced lipid peroxidation products, increased antioxidant enzyme activity, and mitigated jejunal damage in animals with ischemia and reperfusion injury [9, 33, 34]. In this study, IIR rats administered oral post-treatment supplementation of vitamin C plus E showed significantly reduced plasma IL-6 and NOx (Table 4), jejunal TBARS levels (Table 3), and MPO activity (Fig 4C). These results indicate that the combination of water- and fat-soluble antioxidant vitamins protects against IIR injury by alleviating oxidative stress, especially lipid peroxidation reactions, and systemic inflammation.

It is well-known that IIR injury involves a deficiency in the energy necessary to maintain homeostasis and an excess generation of oxygen radicals and inflammatory mediators, leading to subsequent local and systemic complications [3, 5]. Our hypothesis was that post-treatment with a combination of amino acids and antioxidant vitamins might additively improve IIR injury by preserving jejunal mucosal integrity and attenuating oxidative stress and inflammation. We found that oral supplementation with glutamine, citrulline, vitamin C and vitamin E significantly increased the protein and DNA content in the jejunal muscularis (Fig 3B and 3C) and decreased the apoptotic index in the jejunum (Fig 4B) of IIR rats. The results of the 2-way ANOVA indicate that supplementation with glutamine plus citrulline was the main factor that increased jejunal DNA and villus height (Fig 2B), while vitamin C plus E was the main factor that increased jejunal protein. Additionally, supplementation with glutamine plus citrulline and vitamin C plus E were the main factors that decreased the jejunal apoptotic index (Fig 4B). We also demonstrated that a combination of glutamine, citrulline, and vitamins C and E significantly decreased TNF-α, IL-6, IFN-γ, and TBARS in the plasma, as well as TNF-α, IL-6, and TBARS in the jejunum (Tables 3 and 4). Supplementation with glutamine plus citrulline and vitamin C plus E were the main factors that decreased proinflammatory cytokines and TBARS levels in the circulation and jejunum, respectively. These findings demonstrate that oral supplementation with a combination of glutamine, citrulline, and vitamins C and E, administered post-treatment following IIR induction, has additive or complementary effects in preventing jejunal damage, systemic and local inflammation, and lipid peroxidation.

This study has several limitations. The dosages of amino acids and antioxidant vitamins may not be optimal for preventing IIR injury. Due to research funding constraints, we selected doses of glutamine and citrulline that had previously demonstrated a significant attenuation of intestinal damage [8]. Similarly, we chose doses of vitamins C and E that were reported by Kacmaz et al. [16] to eliminate oxygen radicals and prevent peroxidative reactions in IIR animals. Furthermore, we did not investigate the molecular mechanisms underlying the improvement of IIR injury. Further studies could focus on cellular signaling pathways, such as NF-κB, MAPK, TLR4-HMGB1, PKC/p66Shc, AMPK-SIRT-1, NLRP3 inflammasome, Nrf2-ARE, and others, involved in IIR-induced injury [27, 35]. Additionally, the functions of distal organs, including the lung, liver, and kidneys, mostly affected by IIR, were not evaluated. Nevertheless, we observed that supplementation with vitamins C and E significantly decreased the IIR-induced increase in serum GPT, suggesting improved liver function. Exploring the effects of and mechanisms of this combination supplementation in multiple organs following IIR injury would be a worthwhile avenue for future research.

## Conclusions

This study confirms that IIR results in abnormal serological and serum biochemical parameters, elevated inflammatory mediators in the blood and jejunum, identifiable mucosal destruction, and decreased villus height and DNA content in the jejunum. Additionally, IIR increases oxidative stress and apoptosis in the jejunum. Our findings reveal that post-treatment supplementation with glutamine plus citrulline has beneficial effects in preserving jejunal morphology, specifically villus height, and alleviating systemic inflammatory mediators, such as IL-6 and NOx, in IIR rats. Post-treatment supplementation with vitamins C plus E improves liver function and reduces jejunal neutrophil activation. Moreover, a combination supplementation with glutamine, citrulline, and vitamins C and E further decreases circulating lipid peroxidation and jejunal proinflammatory cytokines and apoptosis, while elevating jejunal protein and DNA content in the muscularis. In conclusion, our findings indicate that a combination supplementation of glutamine, citrulline, and antioxidant vitamins may alleviate IIR-induced jejunal damage, local and systemic oxidative stress, and inflammatory response. This post-treatment combination can be used as an adjuvant to attenuate IIR-induced complications.
